# Generation of Rare Sugars by Electrochemical Oxidation of d‑Glucose Using Boron-Doped Diamond Electrode

**DOI:** 10.1021/jacs.4c17553

**Published:** 2025-05-08

**Authors:** Kio Kawakatsu, Sho Usuki, Tiangao Jiang, Naoko Taki, Yuma Uesaka, Haru Togawa, Shanhu Liu, Yasuaki Einaga, Kazuya Nakata

**Affiliations:** † Graduate School of Bio-Applications and Systems Engineering, 155204 Tokyo University of Agriculture and Technology, 2-24-16 Naka-cho, Koganei, Tokyo 184-0012, Japan; ‡ Henan Joint International Research Laboratory of Environmental Pollution Control Materials, Henan Key Laboratory of Polyoxometalate Chemistry, College of Chemistry and Chemical Engineering, 12411 Henan University, Kaifeng 475004, P. R. China; § Department of Chemistry, 12869 Keio University, 3-14-1 Hiyoshi, Yokohama 223-8522, Japan

## Abstract

The electrochemical oxidation of biomass for the production of value-added chemicals represents a promising approach in the field of sustainable chemistry. In this study, we investigated the electrochemical conversion of d-glucose, a biomass-derived compound, using boron-doped diamond (BDD) electrodes under constant applied current (10 mA) or potentials (1.5–3.0 V vs Ag/AgCl). The reaction products were analyzed using high-performance liquid chromatography (HPLC) and liquid chromatography/mass spectrometry (LC/MS) measurements, employing both *p*-aminobenzoic acid ethyl ester (ABEE) and l-tryptophan amide labeling methods to enable characterization. The results demonstrated that the BDD electrodes achieved 95.9% d-glucose degradation and successfully generated various rare sugars, including d-arabinose (0.126 mmol/L), d-erythrose (0.0544 mmol/L), d-glyceraldehyde, and l-glyceraldehyde (combined 0.148 mmol/L). Under identical conditions, Pt electrodes as a control showed only 10.2% d-glucose degradation with significantly lower rare sugar yields. The applied potential significantly influenced the product distribution, with optimal rare sugar production observed at 2.5 V vs Ag/AgCl, reflecting a balance between glucose oxidation and product degradation. Mechanistic studies suggest that the formation of rare sugars involves a series of oxidation and decarboxylation reactions, facilitated by electrochemically generated active species. The superior performance of the BDD electrodes is attributed to their wide potential window, efficient generation of oxidizing species, and unique surface characteristics. This research provides new insights into the electrochemical transformation of biomass-derived compounds and demonstrates the potential for sustainable production of high-value rare sugars, opening avenues for applications in food science, pharmaceuticals, and green chemistry.

## Graphical abstract

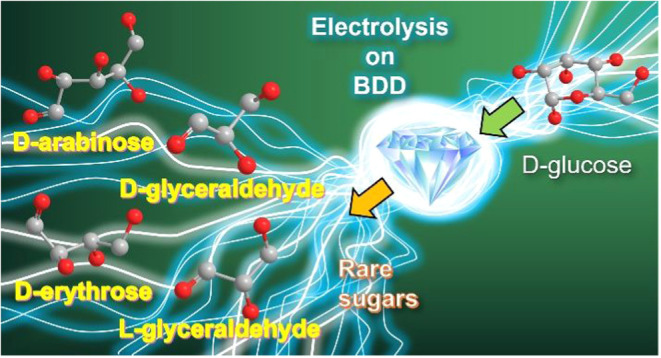

## Introduction

Biomass represents a promising renewable resource for addressing global environmental challenges, particularly for reducing carbon dioxide emissions and achieving sustainable development goals.^1,2^ The utilization of biomass has evolved beyond traditional fuel applications to encompass the production of diverse value-added chemicals.^3^ This transformation is particularly significant for developing sustainable alternatives to petroleum-based chemical processes. Recent advances have demonstrated the versatility of biomass as feedstock for the production of various platform chemicals. Notable examples include 5-hydroxymethylfurfural,^4^ which serves as a key intermediate for biofuel production; gluconic acid^5^ and glucaric acid,^5^ which find applications in pharmaceutical and food industries; and levulinic acid^6^ and formic acid,^7^ which are essential precursors for various industrial chemicals. These biomass-derived compounds are increasingly utilized across multiple sectors, from food additives to raw materials for plastics and rubber production, as well as in the development of eco-friendly preservatives.^8^

Rare sugars represent a unique class of monosaccharides, defined by the International Society of Rare Sugars as “monosaccharides and their derivatives that exist in small quantities in nature”.^9^ The distribution of monosaccharides shows a remarkable disparity: seven types of monosaccharides, known as natural-type monosaccharides, constitute approximately 99.9% of all the sugars found in nature. These abundant monosaccharides include four hexoses (d-glucose, d-fructose, d-mannose, and d-galactose) and three pentoses (d-xylose, l-arabinose, and d-ribose). In contrast, more than 50 distinct types of rare sugars have been identified, collectively accounting for less than 0.1% of total sugar content in natural systems. This striking abundance difference between common and rare sugars has historically limited their availability for research and practical applications.

In contrast, the development of efficient synthetic methods for rare sugars, particularly the establishment of the Izumoring system by Izumori et al. in 2002,^9^ has enabled systematic investigation of their biological activities and potential applications.^10^ Subsequent research has revealed diverse pharmacological properties of various rare sugars, demonstrating their potential value in therapeutic and nutraceutical applications.^11^ d-Allose has demonstrated distinct biological activities, including antioxidant effects through reactive oxygen species scavenging^12^ and inhibition of osteoclast differentiation,^13^ indicating its potential applications in oxidative stress-related conditions and bone metabolism disorders. d-Arabinose has shown promising biological effects, including growth inhibition of nematodes^14^ and improvement of depression-like behavior through modulation of CRTC1 mRNA expression,^15^ suggesting its potential applications in both agricultural pest control and mental health treatment. These diverse biological activities of rare sugars, together with their unique structural features that enable specific biological interactions differ from those of common sugars, suggest their potential value in various applications.

The syntheses of rare sugars can be achieved through enzymatic and chemical approaches, though both methods face significant technical challenges. The industrial-scale enzymatic synthesis faces challenges including high production costs due to expensive cofactors like NADP^+^ and inherently low yields—for example, the conversion of d-glucose to d-allose achieves a maximum theoretical yield of only 2.5% due to thermodynamic equilibrium limitations.^16–18^ Chemical approaches, including carbon chain homologation^19^ and reductive transformations,^20^ also face major limitations: they require energy-intensive conditions with high temperatures (160–200 °C) and pressures (10 MPa),^21^ and the structural complexity of carbohydrates leads to poor selectivity, with reactions like the Lobry de Bruyn-Alberda van Ekenstein transformation typically producing approximately 50 distinct products, severely compromising synthetic efficiency.^18^

The electrochemical oxidation of glucose, a readily available biomass-derived feedstock, has emerged as a promising route for generating diverse value-added compounds. Systematic investigations using various electrode materials have demonstrated that product distribution can be effectively controlled through electrode selection and reaction conditions.^22^ Armstrong et al. demonstrated that Pt/TiO_2_ systems can produce gluconic acid, guluronic acid, and glucono-1,4-lactone.^23^ Hay et al. achieved the formation of rare sugars using graphite electrodes and successfully generated arabinose and erythrose through the electrochemical oxidation of d-glucose.^24^ These studies collectively illustrate the versatility of electrochemical oxidation strategies and demonstrate how careful selection of electrode materials can influence reaction pathways and product distributions.

Boron-doped diamond (BDD) electrodes, synthesized through the incorporation of boron atoms into the diamond lattice, exhibit unique physicochemical and electrochemical properties that distinguish them from conventional electrode materials. The introduction of boron atoms creates p-type semiconductor characteristics, while maintaining the fundamental structural advantages of diamond. The electrochemical characteristics of BDD electrodes include an exceptionally wide potential window and remarkably low background current,^25–27^ properties that arise from their distinctive electronic structure. A distinguishing feature of BDD electrodes is their superior ability to generate powerful oxidizing species during water oxidation processes. These electrodes efficiently produce hydroxyl radicals,^28,29^ ozone^28,29^ and other reactive oxygen species at their surfaces,^28–31^ resulting in enhanced oxidation capabilities. Furthermore, BDD electrodes demonstrate exceptional electrochemical stability due to their sp^3^-hybridized carbon framework,^32^ exhibiting remarkable resistance to surface fouling and maintaining consistent electrochemical response even under demanding conditions. These combined characteristics—wide potential window, efficient generation of oxidizing species, and outstanding stability—have established BDD electrodes as particularly effective for advanced oxidation processes. Their applications range from wastewater treatment to organic synthesis,^33–35^ where their unique properties enable efficient and selective oxidation reactions.

Current methodologies for rare sugar synthesis face significant technical and practical limitations that hinder their widespread industrial implementation. Enzymatic approaches, which offer high selectivity and mild reaction conditions, are constrained by the prohibitive costs associated with both enzymes and essential cofactors, as well as inherently low yields due to unfavorable reaction equilibria. Chemical synthesis routes, particularly those employing oxidizing agents, present challenges in terms of toxicity management and complex product distribution, necessitating elaborate purification procedures. The electrochemical oxidation of glucose has emerged as a promising alternative strategy, and numerous studies have documented the formation of various oxidation products. However, these investigations have primarily focused on the production of organic acids such as gluconic acid and glucaric acid, with limited attention given to the potential formation of rare sugars. BDD electrodes offer unique characteristics in organic oxidation processes, including their wide potential window and superior stability. However, systematic investigations of rare sugar production using BDD electrodes are lacking in the literature. Although BDD electrodes efficiently generate reactive oxygen species, the relationship between this characteristic and formation of rare sugars requires careful investigation. This represents a significant knowledge gap in the understanding of the potential of BDD electrodes for controlled carbohydrate transformations.

In this study, we examined the electrochemical oxidation of d-glucose using BDD electrodes under various conditions, including constant current and controlled potential modes (1.5–3.0 V vs Ag/AgCl). Through product analysis using high-performance liquid chromatography (HPLC) and liquid chromatography–mass spectrometry (LC–MS) with dual labeling strategies (*p*-aminobenzoic acid ethyl ester (ABEE) and l-tryptophan amide), we investigated the formation of rare sugars and elucidated their production pathways. In particular, we focus on understanding the relationship between the electrochemical parameters and product distribution, aiming to provide insights into the factors controlling the formation of valuable rare sugar products. The comparison between the BDD and Pt electrodes under identical conditions allowed us to evaluate the influence of the electrode materials on the reaction outcomes.

## Results and Discussion

Figure 1a shows the time-dependent changes in d-glucose concentration during constant current electrolysis (10 mA). When using the BDD electrode, d-glucose concentration decreased rapidly and continuously, reaching 0.41 mmol L^–1^ after 6 h of electrolysis, corresponding to a decomposition efficiency of 95.9%. In contrast, electrolysis using the Pt electrode under identical conditions resulted in significantly lower d-glucose decomposition, with the concentration decreasing to only 9.01 mmol L^–1^ after 6 h, equivalent to a decomposition efficiency of 10.2%.

**Figure 1:**
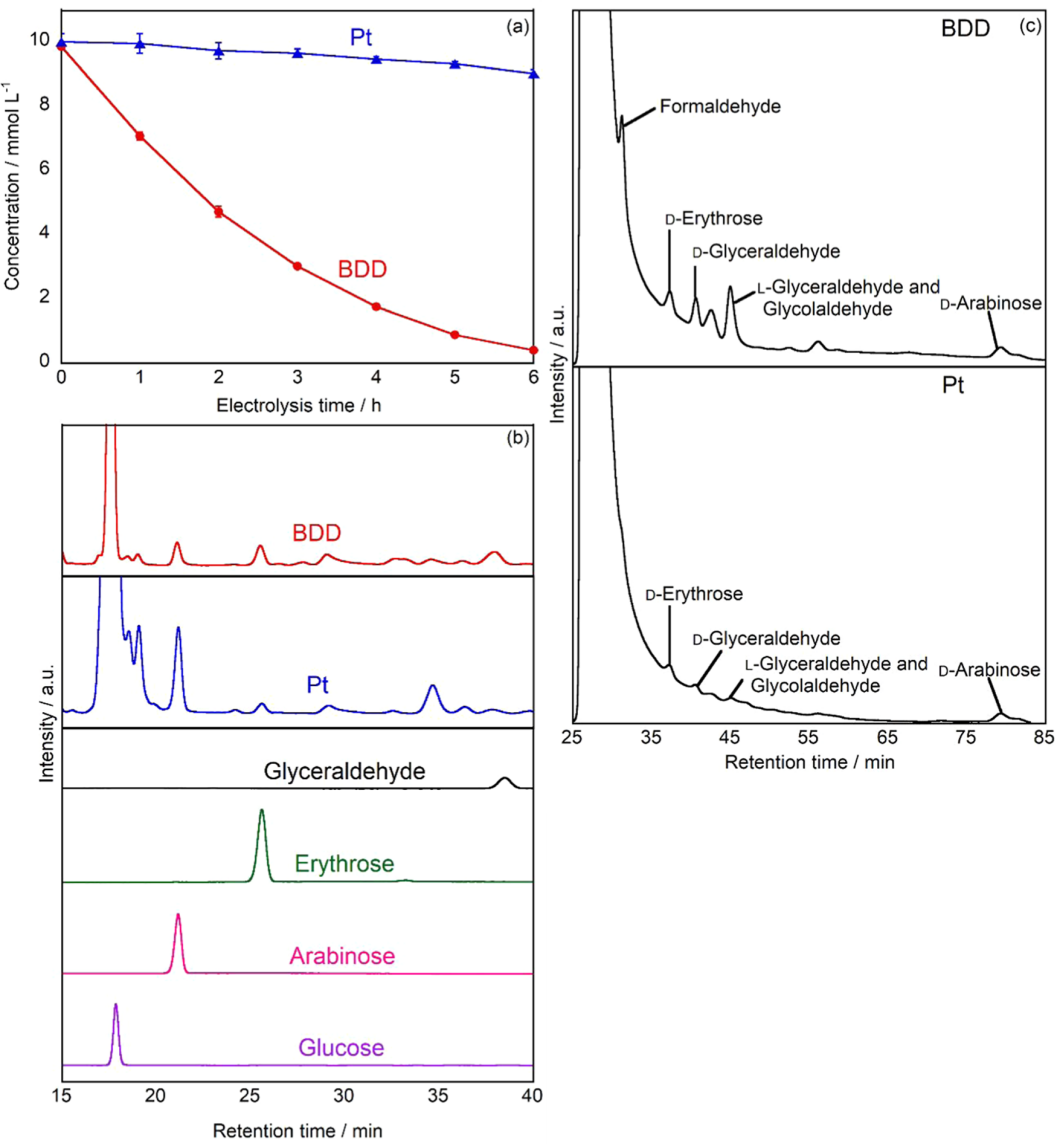
(a) Time-dependent changes in the d-glucose concentration during constant current electrolysis using BDD (red) and Pt (blue) anodes. (b) HPLC chromatograms of the ABEE-labeled sample after 2 h of electrolysis using a BDD (red) and after 6 h of electrolysis using a Pt (blue) electrodes, and standards of glucose (purple), arabinose (pink), erythrose (green), and glyceraldehyde (black). (c) HPLC chromatograms of l-tryptophanamide-labeled sample after 2 h of electrolysis using a BDD electrode and after 6 h of electrolysis using a Pt electrode. Experimental conditions: volume = 20 mL (anode: 10 mL, cathode: 10 mL), electrode area = 18 mm × 18 mm, cathode = Pt, electrolyte = 0.2 mol L^–1^ Na_2_SO_4_, initial concentration of glucose = 10 mmol L^–1^, applied current density = 10 mA cm^–2^.

Qualitative analysis of the reaction products was performed on samples obtained from constant current electrolysis (10 mA) using both BDD and Pt electrodes. Samples were collected after electrolysis for the BDD and Pt electrodes, and then derivatized with ABEE prior to HPLC analysis. HPLC chromatograms of both electrode samples are shown in Figure 1b. For the BDD electrode sample, four major peaks were observed, with R.T. of 17.7, 21.3, 25.6, and 38.3 min. In the case of the Pt electrode sample, similar peaks were detected at R.T. of 17.6, 21.2, 25.6, and 37.8 min. Through comparison with ABEE-labeled standard compounds analyzed under identical conditions, these peaks were identified as glucose, arabinose, erythrose, and glyceraldehyde, respectively.

Molecular weight measurements were performed using LC/MS to further identify the ABEE-labeled products. The observed values were determined by considering the ABEE labeling modification, which can be expressed as [*M* + ABEE – O + H]^+^ = [*M* + 150]^+^, where *M* represents the molecular weight of the original sugar. For the products obtained using the BDD electrode, the peak at R.T. = 21.3 min exhibited *m*/*z* = 300 (Figure S1a), corresponding to the calculated weight of the ABEE-labeled arabinose. Similarly, the peaks at R.T. 25.6 and 38.3 min showed *m*/*z* values of 270 and 240, respectively (Figure S1b,c), consistent with the calculated weights of ABEE-labeled erythrose and glyceraldehyde.

For the Pt electrode products, the peak at R.T. = 21.2 min yielded *m*/*z* = 300 (Figure S1d), confirming the presence of ABEE-labeled arabinose. The peak at R.T. = 25.6 min, tentatively assigned as erythrose based on the retention time, could not be confirmed by mass spectrometry because its concentration was below the detection limit.

Monosaccharides exist as d- and l-enantiomers, which exhibit identical physicochemical properties and are thus difficult to separate using conventional HPLC analysis. Therefore, we employed an enantiomeric analysis method utilizing l-tryptophan amide labeling, as reported by Kodama et al.^36^ This method enables the separation of monosaccharide enantiomers through the formation of diastereomeric derivatives, which can be resolved on a reverse-phase column because of their distinct hydrophobic properties. While previous studies have employed phosphate-based mobile phases, we modified the method to use an ammonium acetate-based mobile phase to enable LC/MS analysis.

HPLC analysis of l-tryptophan amide-labeled products from the BDD electrode electrolysis revealed peaks at R.T. of 31.2, 37.3, 40.6, 45.0, and 79.4 min, which were identified through comparison with standard compounds such as formaldehyde, d-erythrose, d-glyceraldehyde, l-glyceraldehyde, glycolaldehyde, and d-arabinose, respectively (Figure 1c).

For the Pt electrode products, the chromatogram exhibited peaks at R.T. of 37.3, 40.6, 45.1, and 79.3 min, corresponding to d-erythrose, d-glyceraldehyde, l-glyceraldehyde and glycolaldehyde, and d-arabinose, respectively (Figure 1c). Notably, this analysis method enabled the detection of both d-glyceraldehyde and l-glyceraldehyde, which were not distinguished in the ABEE labeling analysis.

To further identify the l-tryptophan amide-labeled products, molecular weight measurements were performed using LC/MS. The observed values were determined considering the l-tryptophan amide labeling modification, which can be expressed as [*M* + l-tryptophan amide – O + H]^+^ = [*M* + 188]^+^, where *M* represents the molecular weight of the original sugar. For the products obtained using the BDD electrode, the peak at R.T. = 79.4 min exhibited *m*/*z* = 338 (Figure S2a), corresponding to the calculated weight of the labeled d-arabinose. Similarly, the peaks at R.T. 37.3 and 40.6 min showed *m*/*z* values of 308 and 278, respectively (Figure S2b,c), consistent with the calculated weights of labeled d-erythrose and d-glyceraldehyde. The peak at R.T. = 31.2 and 45.0 min yielded *m*/*z* values of 218, 248, and 278 (Figure S3d,e), corresponding to labeled formaldehyde, glycolaldehyde and l-glyceraldehyde, respectively.

For the Pt electrode products, analogous LC/MS analysis revealed *m*/*z* values of 338, 308, and 278 for peaks at R.T. 79.3, 37.3, and 40.6 min (Figure S3a–c), confirming the presence of labeled d-arabinose, d-erythrose, and d-glyceraldehyde, respectively. The peak at R.T. = 45.1 min exhibited *m*/*z* values of 248 and 278, respectively, indicating the presence of both labeled glycolaldehyde and l-glyceraldehyde (Figure S3d).

Previous studies have demonstrated the formation of organic acids during glucose electrolysis, with Liu et al. reporting gluconic acid production and Chen et al. identifying formic acid production.^37,38^ To verify the formation of these organic acids in this system, HPLC analysis was performed on samples collected after electrolysis using BDD and Pt electrodes. Figure 2 shows the HPLC chromatograms of these samples along with standard solutions of gluconic acid and formic acid. For the BDD electrode sample, two peaks were observed at R.T. of 11.8 and 17.3 min, which corresponded to the R.T. of standard gluconic acid and formic acid, respectively. Similarly, in the Pt electrode sample (Figure 2), peaks were detected at identical R.T. (11.8 and 17.3 min), confirming the presence of both gluconic acid and formic acid.

**Figure 2:**
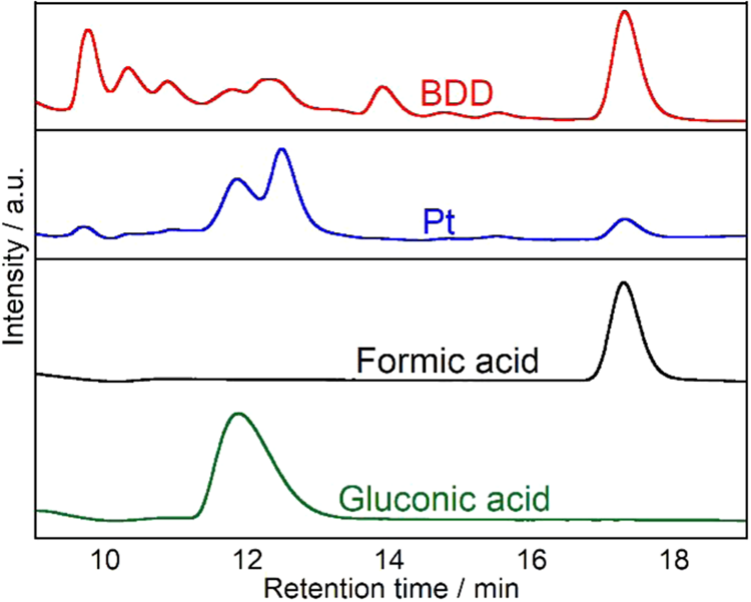
HPLC chromatograms of the sample after 2 h of electrolysis using a BDD electrode (red) and after 6 h of electrolysis using a Pt electrode (blue), and gluconic acid (green) and formic acid (black) standards. Experimental conditions: volume = 20 mL (anode: 10 mL, cathode: 10 mL), electrode area = 18 mm × 18 mm, cathode = Pt, electrolyte = 0.2 mol L^–1^ Na_2_SO_4_, initial concentration of glucose = 10 mmol L^–1^, applied current density = 10 mA cm^–2^.

The time-dependent changes in product concentrations during d-glucose electrolysis were monitored using ABEE-labeled HPLC analysis. Figure 3a shows the concentration profiles of rare sugars produced using the BDD electrode. d-Arabinose and d-erythrose concentrations reached their maxima at 2 h of electrolysis, with values of 0.126 and 0.0544 mmol L^–1^, respectively. The concentration of d, l-glyceraldehyde peaked at 3 h, reaching 0.148 mmol L^–1^. After these maximum points, the concentrations of all products gradually decreased. The observed decrease in product concentrations after reaching their maximum values can be attributed to the further electrochemical oxidation of these products. The rare sugars formed during the initial stages of electrolysis underwent electrochemical decomposition to form other products, leading to the observed concentration decline after 2–3 h.

**Figure 3:**
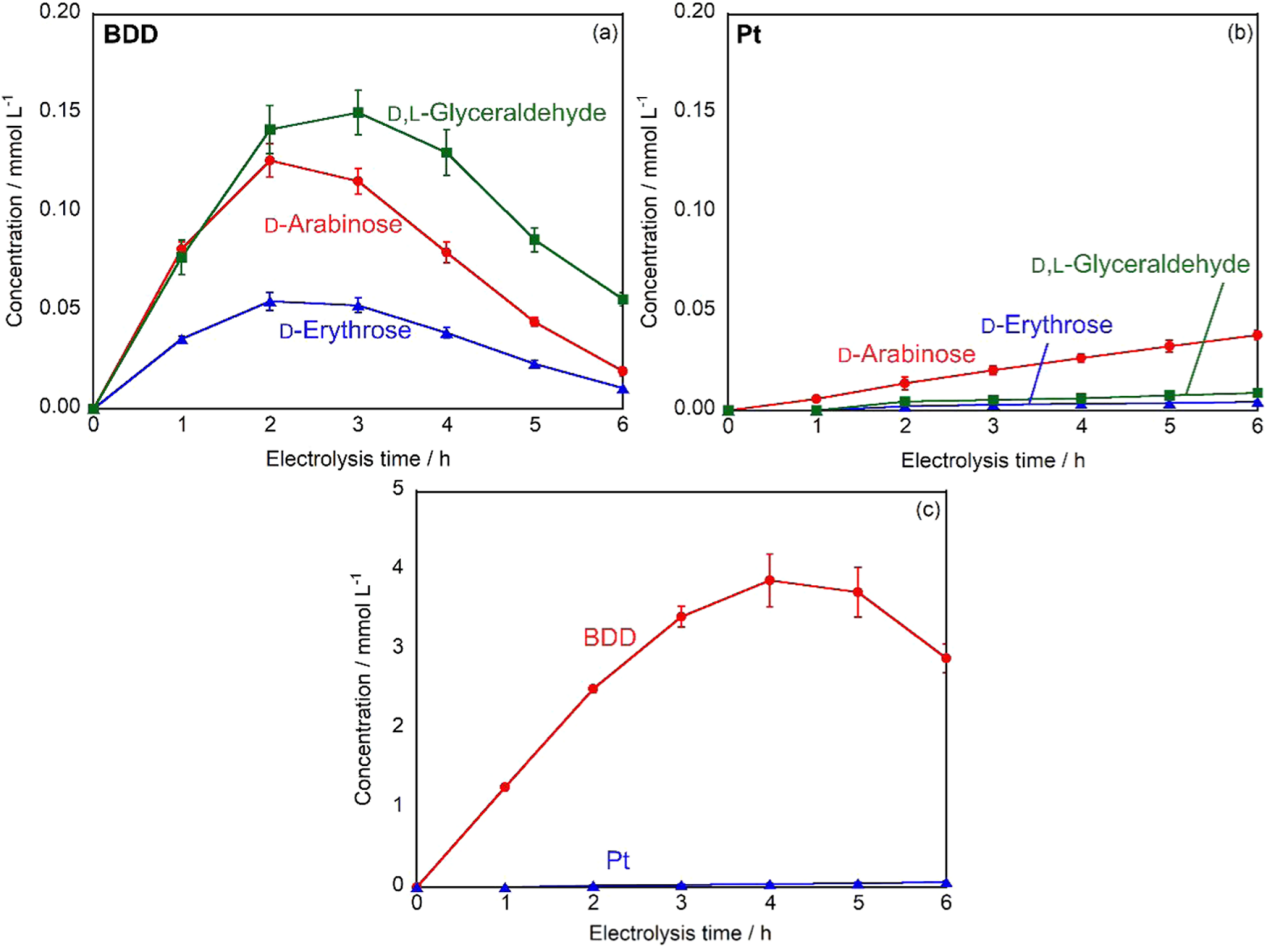
Time-dependent changes in concentrations of ABEE-labeled products during constant current electrolysis using (a) BDD anode and (b) Pt anode: d-arabinose (red), d-erythrose (blue), and d,l-glyceraldehyde (green). (c) Time-dependent changes in formic acid concentration during constant current electrolysis using BDD (red) and Pt (blue) electrodes. Experimental conditions: volume = 20 mL (anode: 10 mL, cathode: 10 mL), electrode area = 18 mm × 18 mm, cathode = Pt, electrolyte = 0.2 mol L^–1^ Na_2_SO_4_, initial concentration of glucose = 10 mmol L^–1^, applied current density = 10 mA cm^–2^.

In contrast, electrolysis using the Pt electrode (Figure 3b) showed markedly different behavior. The concentrations of all products increased gradually throughout the 6 h electrolysis period, with no distinct peak values observed. After 6 h, the final concentrations were 0.0381 mmol L^–1^ for d-arabinose, 0.00435 mmol L^–1^ for d-erythrose, and 0.00880 mmol L^–1^ for d, l-glyceraldehyde. These values were significantly lower than those obtained with the BDD electrode, representing only 30.2, 8.0, and 5.9% of the maximum concentrations achieved with BDD for d-arabinose, d-erythrose, and d, l-glyceraldehyde, respectively.

The time-dependent changes in organic acid concentrations were monitored during electrolysis. While gluconic acid was detected, its accurate quantification was hindered by peak overlap with unidentified products in the HPLC analysis. Therefore, we focused on the quantitative analysis of formic acid formation. Figure 3c shows the time course of formic acid concentration for both BDD and Pt electrodes. Using the BDD electrode, formic acid concentration increased rapidly from the onset of electrolysis, reaching a maximum concentration of 3.96 mmol L^–1^ at 4 h. Subsequently, the concentration decreased, following a trend similar to that observed for the rare sugar products. This decrease can be attributed to the further electrochemical oxidation of formic acid to other products. In contrast, with the Pt electrode, formic acid was first detected after 4 h of electrolysis, and its concentration increased slowly to reach only 0.0752 mmol L^–1^ after 6 h, which is approximately 1.9% of the maximum concentration achieved with the BDD electrode.

Under constant current conditions (10 mA), the BDD electrode demonstrated superior performance compared with the Pt electrode in terms of both d-glucose decomposition and product formation. The BDD electrode achieved 95.9% d-glucose degradation after 6 h of electrolysis, while the Pt electrode showed only 10.2% degradation. While the current is the same for both electrodes, this demonstrates the superior oxidation capability of BDD, which can achieve more effective glucose conversion despite operating at higher potentials than Pt. This enhanced activity of the BDD electrode was also reflected in product formation, where maximum concentrations of rare sugars were significantly higher: d-arabinose (0.126 mmol L^–1^ vs 0.0381 mmol L^–1^), d-erythrose (0.0544 mmol L^–1^ vs 0.00435 mmol L^–1^), and d, l-glyceraldehyde (0.148 mmol L^–1^ vs 0.00880 mmol L^–1^). Similarly, formic acid production was substantially higher with the BDD electrode, reaching a maximum concentration of 3.96 mmol L^–1^, compared to 0.0752 mmol L^–1^ with the Pt electrode. These results clearly demonstrate that the BDD electrode is more effective for the electrochemical conversion of d-glucose to rare sugars and other valuable products under constant current conditions.

Following the constant current experiments, constant potential electrolysis was conducted to elucidate the relationship between applied potential and product selectivity, as the electrode potential is a crucial parameter that directly influences the oxidation process. The effect of applied potential was investigated using a BDD electrode at potentials ranging from 1.5 to 3.0 V vs Ag/AgCl. An electrolyte of 0.2 mol L^–1^ sodium sulfate was used with Pt as the cathode, and 10 mmol L^–1^ glucose was electrolyzed for 6 h. Figure 4 shows the time-dependent changes in d-glucose concentration during constant potential electrolysis. The decomposition efficiency of d-glucose exhibited a clear potential dependence, with higher applied potentials leading to more rapid decomposition. At 1.5 V vs Ag/AgCl, the d-glucose concentration decreased moderately to 9.93 mmol L^–1^ after 6 h, corresponding to a decomposition efficiency of 0.7%. The decomposition rate progressively increased with applied potential, and at 3.0 V vs Ag/AgCl, d-glucose was completely decomposed within 6 h, with the concentration falling below the detection limit.

**Figure 4:**
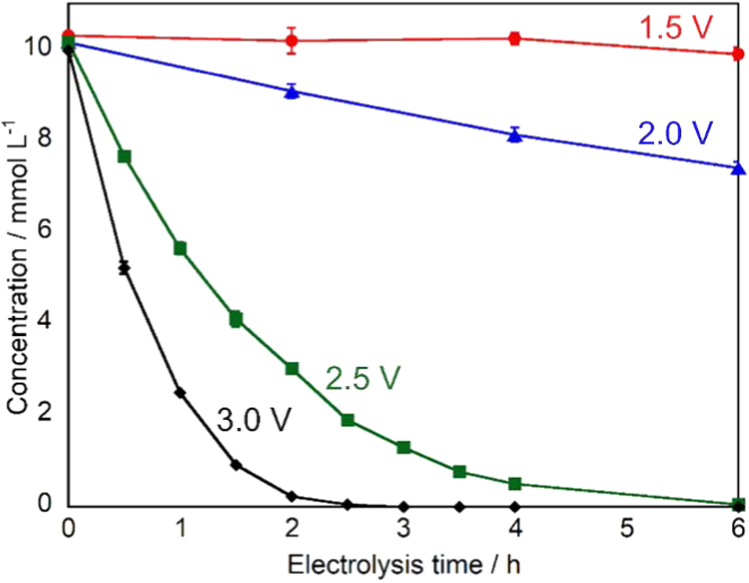
Time-dependent changes in d-glucose concentration during constant potential electrolysis using BDD electrode at different applied potentials: 1.5 V (red), 2.0 V (blue), 2.5 V (green), and 3.0 V (black) vs Ag/AgCl. Experimental conditions: volume = 20 mL (anode: 10 mL, cathode: 10 mL), electrode area = 18 mm × 18 mm, cathode = Pt, electrolyte = 0.2 mol L^–1^ Na_2_SO_4_, initial concentration of glucose = 10 mmol L^–1^.

The time-dependent concentration changes of rare sugars produced during constant potential electrolysis were investigated using the BDD electrode. Figure 5a shows the concentration profiles of d-arabinose at various applied potentials (1.5–3.0 V vs Ag/AgCl). At lower potentials (1.5 and 2.0 V vs Ag/AgCl), d-arabinose concentration increased steadily throughout the 6 h electrolysis period, reaching maximum values of 0.0155 and 0.0505 mmol L^–1^, respectively. In contrast, at higher potentials (2.5 and 3.0 V vs Ag/AgCl), the concentration profiles exhibited distinct maxima at 1.5 h (0.0929 mmol L^–1^) and 0.5 h (0.0180 mmol L^–1^), respectively, followed by gradual decreases.

**Figure 5:**
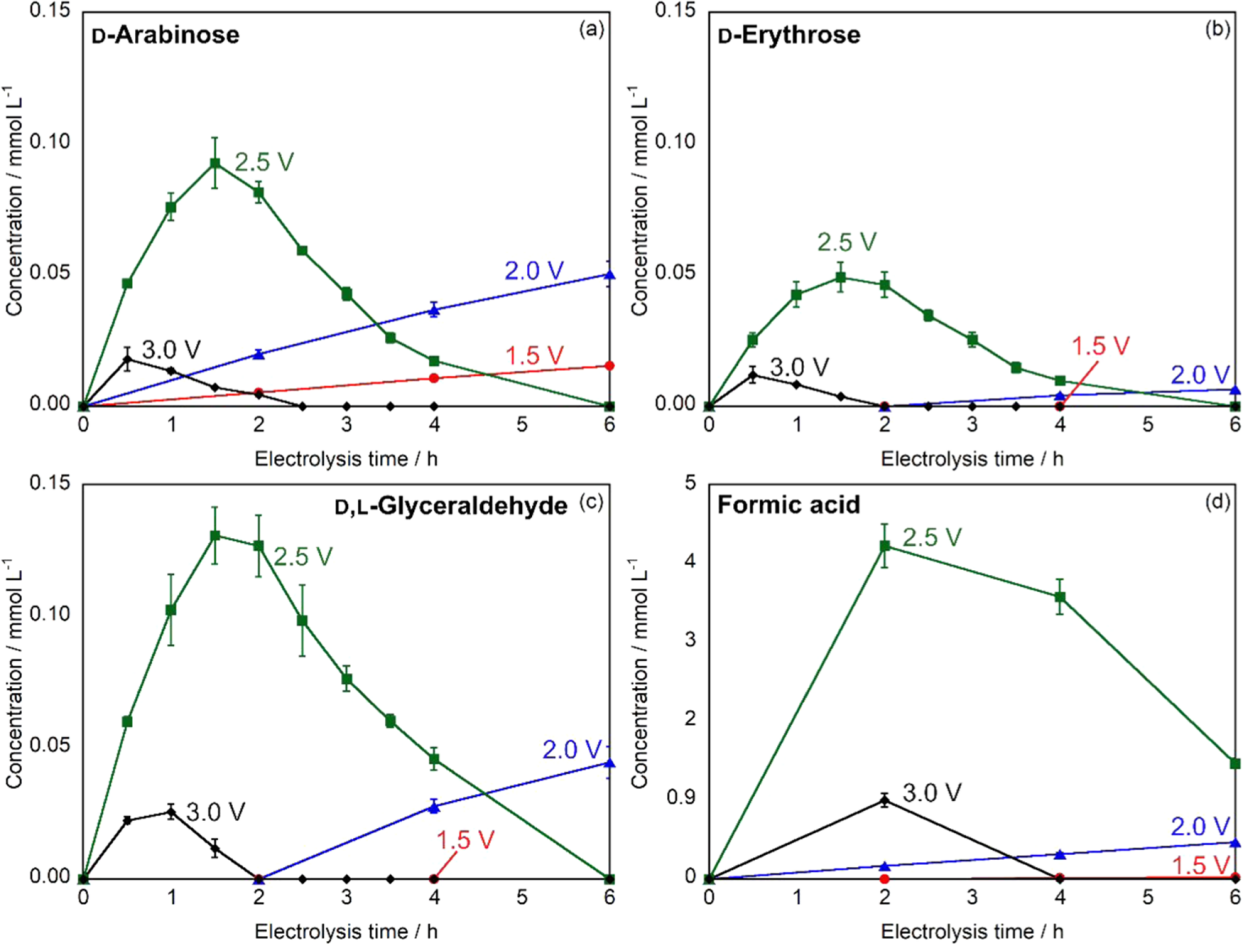
Time-dependent changes in (a) d-arabinose, (b) d-erythrose, (c) d,l-glyceraldehyde and (d) formic acid concentration during constant potential electrolysis using BDD electrode at different applied potentials: 1.5 V (red), 2.0 V (blue), 2.5 V (green), and 3.0 V (black) vs Ag/AgCl. Experimental conditions: volume = 20 mL (anode: 10 mL, cathode: 10 mL), electrode area = 18 mm × 18 mm, cathode = Pt, electrolyte = 0.2 mol L^–1^ Na_2_SO_4_, initial concentration of glucose = 10 mmol L^–1^.

The formation of d-erythrose demonstrated similar potential-dependent behavior (Figure 5b). No d-erythrose was detected at 1.5 V vs Ag/AgCl, while at 2.0 V vs Ag/AgCl, the concentration increased continuously to reach 0.00650 mmol L^–1^ after 6 h. At 2.5 and 3.0 V vs Ag/AgCl, maximum concentrations of 0.0492 and 0.0120 mmol L^–1^ were observed at 1.5 and 0.5 h, respectively, before declining.

The combined concentration of d- and l-glyceraldehyde showed similar trends (Figure 5c). No glyceraldehyde formation was observed at 1.5 V vs Ag/AgCl, whereas at 2.0 V vs Ag/AgCl, the concentration increased steadily to 0.0526 mmol L^–1^ over 6 h. At higher potentials of 2.5 and 3.0 V vs Ag/AgCl, the maximum concentrations of 0.140 and 0.0255 mmol L^–1^ were reached at 1.5 and 1.0 h, respectively, followed by a decrease.

The concentration decreases observed at higher potentials can be attributed to the further electrochemical oxidation of the produced rare sugars to other products, similar to the behavior observed under constant current conditions. These results indicate that the applied potential significantly influences both the formation rate and stability of rare sugar products.

The formation of organic acids during constant potential electrolysis was monitored by HPLC analysis. Although gluconic acid was detected, its quantification was not feasible because of peak overlap with unidentified products. Therefore, we focused on the quantitative analysis of formic acid production. Figure 5d illustrates the time-dependent changes in formic acid concentration at applied potentials ranging from 1.5 to 3.0 V vs Ag/AgCl using the BDD electrode. At lower potentials (1.5 and 2.0 V vs Ag/AgCl), formic acid concentration increased steadily throughout the 6 h electrolysis period, reaching final concentrations of 0.0230 and 0.423 mmol L^–1^, respectively. In contrast, at higher potentials (2.5 and 3.0 V vs Ag/AgCl), the concentration profiles exhibited maxima at 2.0 h of electrolysis, reaching 3.81 and 0.904 mmol L^–1^, respectively, followed by gradual decreases. The highest formic acid concentration was achieved at 2.5 V vs Ag/AgCl, which was approximately 165 times higher than that obtained at 1.5 V vs Ag/AgCl. The subsequent decrease in formic acid concentration at higher potentials can be attributed to its further electrochemical oxidation to other products. These results demonstrated that the applied potential significantly influenced both the formation rate and stability of formic acid, with an optimal potential of 2.5 V vs Ag/AgCl for maximum production.

The applied potential significantly influenced both d-glucose decomposition and product formation at the BDD electrode. At lower potentials (1.5 and 2.0 V vs Ag/AgCl), the relatively slow d-glucose decomposition and limited product formation can be attributed to insufficient generation of electrochemically active species. These active species, including hydroxyl radicals, ozone, hydrogen peroxide, sulfate radicals, and peroxodisulfate ions,^28–31^ are essential for the oxidation of d-glucose and subsequent formation of rare sugars and organic acids. The low concentrations of these species at lower potentials resulted in reduced reaction rates and product yields.

Conversely, at the highest potential (3.0 V vs Ag/AgCl), despite complete d-glucose decomposition, the product yields were notably lower than those obtained at 2.5 V vs Ag/AgCl. For instance, the maximum concentrations of d-arabinose (0.0180 mmol L^–1^), d-erythrose (0.0120 mmol L^–1^), and d, l-glyceraldehyde (0.0255 mmol L^–1^) at 3.0 V were significantly lower than those at 2.5 V (0.0929, 0.0492, and 0.140 mmol L^–1^, respectively). This phenomenon can be explained by the excessive generation of active species at higher potentials, leading to rapid oxidation of not only d-glucose but also the formed products. This resulted in decreased product yields through subsequent decomposition reactions.

The optimal potential of 2.5 V vs Ag/AgCl appears to provide a balanced generation of active species, facilitating efficient d-glucose oxidation while maintaining relatively stable product concentrations. These findings demonstrate the crucial role of potential control in optimizing the selective formation of desired products during electrochemical d-glucose oxidation.

The superior performance of the BDD electrode compared to that of the Pt electrode for d-glucose decomposition and product formation can be attributed to two primary factors: the difference in the effective potential during electrolysis and the distinct electrochemical characteristics of each electrode material. The potential profiles during constant current electrolysis (10 mA) are shown in Figure S4. The BDD electrode maintained an average potential of 2.4 V vs Ag/AgCl, which was 0.5 V higher than that of the Pt electrode (1.9 V vs Ag/AgCl). This higher effective potential at the BDD electrode surface promotes more efficient oxidation reactions, contributing to enhanced d-glucose decomposition and product formation. More significantly, the fundamental difference in electrode surface characteristics plays a crucial role in their catalytic activity. The BDD electrode, classified as an inactive electrode, allows hydroxyl radicals to physically adsorb on its surface without chemical interaction.^28,39,40^ These physically adsorbed hydroxyl radicals maintain their high oxidation potential (2.73 V vs NHE) and directly participate in oxidation reactions. Previous studies have shown that direct oxidation of glucose on a BDD electrode may occur at lower potentials, but when potentials exceeding 2.4 V are applied, indirect oxidation becomes the predominant mechanism.^41^ Since our experiments applied a potential of 2.4 V vs Ag/AgCl to the BDD electrode, we believe that indirect oxidation was the primary mechanism, consistent with existing literature. BDD electrodes are considered inactive electrodes where the direct anodic reaction primarily generates hydroxyl radicals rather than facilitating direct electron transfer with the substrate. Based on these observations, we conclude that glucose oxidation on the BDD electrode proceeded predominantly through an indirect oxidation pathway mediated by generated hydroxyl radicals. In contrast, the Pt electrode, an active electrode, chemically interacts with hydroxyl radicals to form platinum oxide species.^28,39,40,42^ These surface-bound oxide species exhibit lower oxidation potential than free hydroxyl radicals, resulting in decreased oxidation efficiency. Furthermore, the oxygen evolution characteristics of these electrodes affect their oxidation efficiency. The Pt electrode, known for its lower oxygen overpotential compared to BDD, promotes the oxygen evolution reaction at lower potentials.^43^ This competing reaction reduces the efficiency of d-glucose oxidation by consuming part of the applied current. Conversely, the higher oxygen overpotential of the BDD electrode suppressed oxygen evolution, allowing more efficient utilization of the applied current for d-glucose oxidation and product formation. These combined factors explain the observed differences in performance: the BDD electrode achieved 95.9% d-glucose decomposition and significantly higher product concentrations (e.g., 3.96 mmol/L formic acid), while the Pt electrode showed only 10.2% decomposition and lower product yields (e.g., 0.0752 mmol L^–1^ formic acid).

To evaluate the electrocatalytic behavior of BDD and Pt electrodes toward glucose oxidation, cyclic voltammetry measurements were conducted (Figure S5). The voltammograms revealed slight differences in onset potential between the two electrode materials. The Pt electrode exhibited glucose oxidation onset at approximately 1.5 V vs Ag/AgCl, whereas the BDD electrode initiated glucose oxidation at higher potentials. The difference in onset potential results in distinct electrochemical operating regimes when both systems are maintained at identical current density (10 mA cm^–2^) during electrolysis. The BDD electrode functions efficiently within a higher potential region, which enables its superior oxidative capabilities through hydroxyl radical generation mechanisms. The BDD electrode achieved 95.9% d-glucose degradation after 6 h of electrolysis, while the Pt electrode showed only 10.2% degradation. This demonstrates the superior oxidation capability of BDD, which can achieve more effective glucose conversion at its characteristic operating potential. The distinct voltammetric behavior correlates with the fundamentally different reaction mechanisms occurring at each electrode surface - hydroxyl radical-mediated oxidation at BDD versus direct surface-catalyzed oxidation at Pt.

To assess the efficiency of electrode processes, we calculated the Faradaic efficiencies for both electrode systems as shown in Table S1. Although absolute values appear modest due to competing oxygen evolution reactions, the relative performance difference between electrodes remains significant. For rare sugar production, the BDD electrode achieved Faradaic efficiencies of 0.6% for d-arabinose and d-erythrose, and 0.2% for d,l-glyceraldehyde, while the Pt electrode yielded merely 0.05% for d-arabinose with undetectable amounts of d-erythrose and d,l-glyceraldehyde. These lower Faradaic efficiency values for rare sugars reflect the fundamental challenge in electrochemical sugar conversion: the water oxidation reaction (oxygen evolution) occurs at potentials similar to those required for sugar transformation, inevitably consuming a substantial portion of the supplied current. Despite this inherent limitation, the BDD electrode demonstrates remarkable selectivity toward valuable rare sugar products compared to conventional Pt electrodes, with efficiency values 12 times higher for d-arabinose and significantly superior for other target compounds. Calculating accurate Faradaic efficiencies for these sugar conversion reactions presents additional challenges due to complex reaction pathways. Glucose oxidation at BDD electrodes proceeds predominantly through hydroxyl radicals (H_2_O → OH^•^ + H^+^ + e^–^), which can initiate multiple competing pathways. For example, glucose conversion to arabinose can produce either formic acid (C_6_H_12_O_6_ + O_2_ + OH^•^ → C_5_H_10_O_5_ + HCOOH + OH^•^) or carbon dioxide (C_6_H_12_O_6_ + O_2_ + OH^•^ → C_5_H_10_O_5_ + CO_2_ + H_2_O + OH^•^). Formic acid and carbon dioxide production are particularly complex, as they can be generated during multiple conversion steps with different electron requirements depending on the reaction pathway. For the purpose of estimation, the Faradaic efficiencies presented in Table S1 were calculated using simplified assumptions about electron transfer requirements: 1-electron for arabinose, 2-electron for erythrose, 3-electron for glyceraldehyde, and 1-electron for formic acid, gluconic acid, and CO_2_. Given the complexity of the actual reaction pathways, particularly for formic acid and CO_2_ formation where 1 to 6 electron processes may occur, these values should be understood as reflecting a simplified representation of the electrochemical processes rather than definitive Faradaic efficiencies. The Faradaic efficiency for carbon dioxide showed 43% for BDD and remained below detection limits for Pt. The high carbon dioxide Faradaic efficiency with BDD likely results from powerful hydroxyl radicals oxidizing not only glucose but also trace organic materials in the electrolysis cell. The combined Faradaic efficiency of 47% for BDD compared to merely 0.8% for Pt demonstrates markedly different current utilization patterns. These results provide strong evidence supporting our proposed mechanism wherein BDD electrodes facilitate glucose oxidation primarily via hydroxyl radical intermediates, offering a promising approach for the selective electrochemical production of valuable rare sugars despite the competing oxygen evolution reaction.

The carbon balance distribution during glucose electrolysis revealed differences between BDD and Pt electrodes (Tables S2 and S3). With BDD electrodes, sugars predominated during initial electrolysis stages, then diminished substantially after 6 h. Organic acids, including gluconic acid and formic acid, constituted approximately 8–20% across all electrolysis times, while the majority of remaining carbon transformed into CO_2_. The carbon balance exceeding 100% with BDD electrodes results from their efficient generation of hydroxyl radicals. These powerful oxidizing species decompose not only glucose but potentially also trace organic substances in the electrolysis cell, contributing additional CO_2_. Regarding CO_2_ production, the fact that CO_2_ remained below detection limits in the Pt electrode experiments confirms that the high CO_2_ values observed with the BDD electrode represent actual electrode reaction products rather than external contamination. With Pt electrodes, 90.1% of starting material remained as glucose after 6 h, with gluconic acid (5.8%) representing the primary oxidation product. Small quantities of other sugars were also detected. These carbon balance distributions demonstrate fundamentally different reaction mechanisms between BDD and Pt electrodes—extensive C–C bond cleavage with BDD versus selective functional group oxidation with Pt.

Compared to Pt electrodes, BDD electrodes demonstrated superior performance for the oxidation of d-glucose to rare sugars. It is also important to compare BDD with other carbon-based electrodes, particularly glassy carbon. When we conducted experiments using glassy carbon as an anode under identical conditions to this study, we observed significant degradation of the electrode itself. Glassy carbon consists predominantly of sp^2^ carbon structures and undergoes dissolution when exposed to potentials above the oxygen evolution potential. This inherent instability makes glassy carbon unsuitable for producing rare sugars that have high potential for future use as pharmaceuticals or food additives, where human safety considerations are paramount. The exceptional stability of BDD electrodes, attributed to their sp^3^-hybridized carbon framework, represents a significant advantage for rare sugar production applications.

The quality of boron-doped diamond significantly influences the electrode performance in terms of glucose conversion and product selectivity. In this study, BDD with B/C = 0.5% was employed to achieve optimal electrical conductivity. Lower boron doping concentrations would reduce electrode conductivity, potentially decreasing both glucose decomposition efficiency and rare sugar yields. Additionally, the presence of sp^2^ carbon in the diamond structure affects electrocatalytic behavior by providing active sites that lower the oxygen evolution potential and facilitate direct oxidation reactions. However, excessive sp^2^ carbon content may cause the BDD to behave more like conventional active electrodes, potentially resulting in decreased Faradaic efficiency and reduced catalytic activity due to surface adsorption phenomena.

It is worth noting that while alkaline electrolytes are known to enhance the activity of Pt electrodes for sugar oxidation, we specifically chose sodium sulfate as the electrolyte due to its neutral pH and high safety profile. Alkaline solutions pose challenges for rare sugar production due to their tendency to promote sugar isomerization reactions and their lower safety profile for potential pharmaceutical and food additive applications. Sodium sulfate, being widely used in pharmaceuticals and food additives, aligns with our focus on developing environmentally benign and safe processes for rare sugar production.

The electrochemical oxidation of d-glucose (hexose, having six carbon atoms, C6) resulted in the formation of various products with shorter carbon chains. These include rare sugars such as d-arabinose (pentose, C5), d-erythrose (tetrose, C4), and d, l-glyceraldehyde (triose, C3), as well as organic acids (gluconic acid and formic acid) and aldehydes (glycolaldehyde and formaldehyde). The product distribution suggested multiple reaction pathways involving oxidation, decarboxylation, and carbon–carbon bond cleavage.

A primary reaction pathway appears to proceed through gluconic acid formation (Figure 6). In this pathway, d-glucose initially undergoes oxidation to form gluconic acid, which subsequently undergoes decarboxylation to produce d-arabinose. This mechanism is supported by the work of Chou et al.^44^ who demonstrated arabinose formation through electrochemical oxidation of sodium gluconate. Following similar oxidation and decarboxylation steps, d-arabinose may be converted to d-erythrose, and subsequently to d-glyceraldehyde.

**Figure 6:**
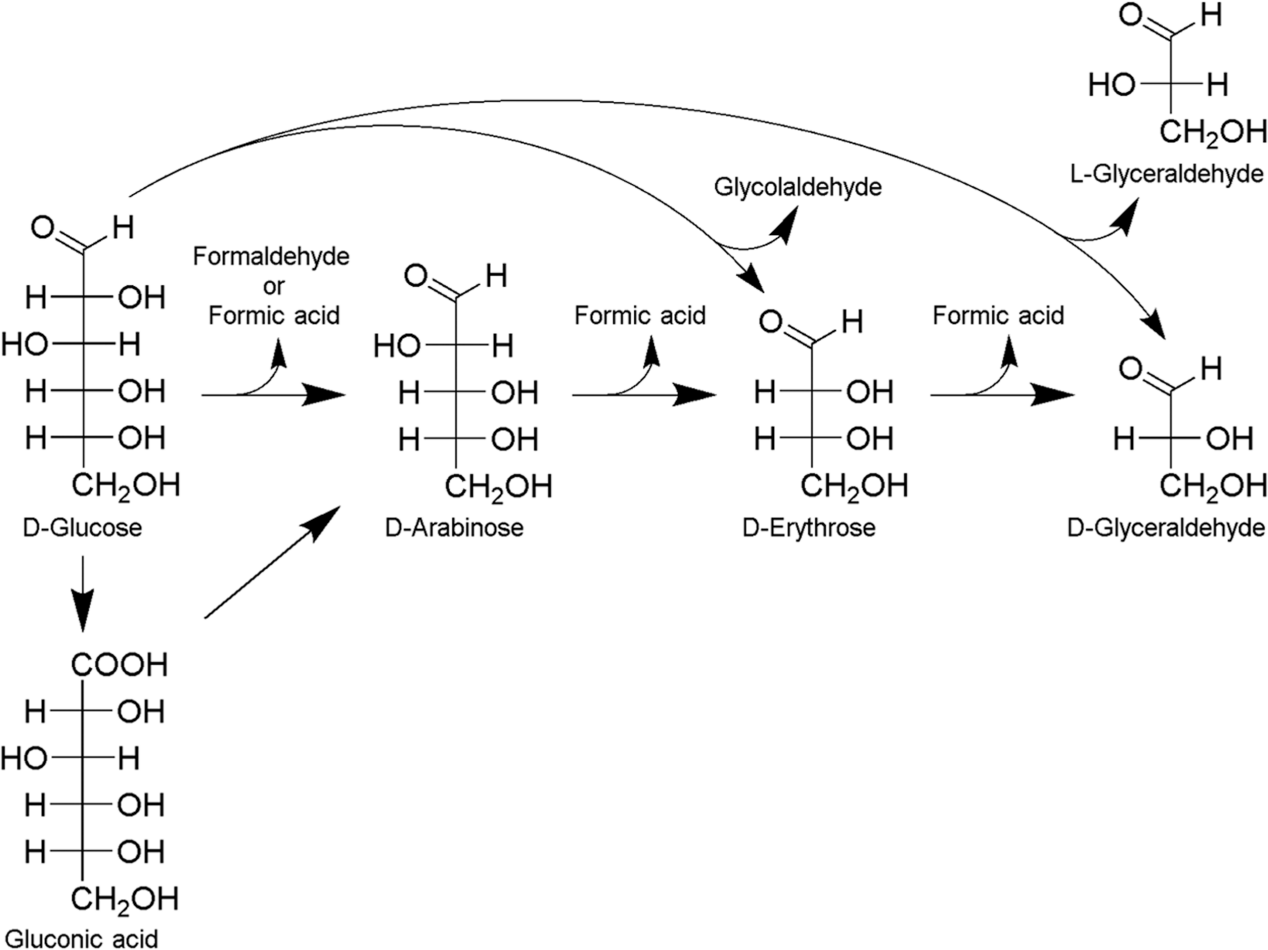
Presumable reaction pathway for the electrochemical conversion of d-glucose.

Another significant pathway involves C1–C2 bond cleavage of d-glucose (Figure 6), leading to the simultaneous formation of d-arabinose and formic acid. This observation aligns with findings by Van der Ham et al. and Moggia et al., who reported similar decarboxylation reactions during glucose electrooxidation at high potentials.^22,45^ Through analogous mechanisms, sequential decarboxylation reactions can produce d-erythrose from d-arabinose, and d-glyceraldehyde from d-erythrose.

Alternative carbon–carbon bond cleavage pathways were also identified. The C2–C3 bond cleavage of d-glucose (Figure 6) directly produces d-erythrose and glycolaldehyde. Additionally, the detection of both d- and l-glyceraldehyde suggests a pathway involving C3–C4 bond cleavage (Figure 6) because l-glyceraldehyde cannot be formed through the sequential decarboxylation of d-glucose.

These oxidation and decarboxylation reactions are likely mediated by both direct electron transfer at the electrode surface and electrochemically generated active species. Under our experimental conditions, five types of active species may be generated through the following reactions (1) to (8)^28–31^

1 M+H2O→M(OH•)+H++e−

2 $${3H}_{2}O\to O_{3}+6H^{+}+6e^{-}$$

3 $${O_{2}+H}_{2}O\to O_{3}+{2H}^{+}+{2e}^{-}$$

4 2OH•→H2O2

5 M(OH•)+SO42−→M(SO4•−)+OH−•

6 $$M\left({SO}_{4}^{•-}\right)+SO_{4}^{2-}\to M+S_{2}O_{8}^{2-}+e^{-}$$

7 $$2SO_{4}^{2-}\to S_{2}O_{8}^{2-}+{2e}^{-}$$

8 $$SO_{4}^{•-}+SO_{4}^{•-}\to S_{2}O_{8}^{2-}$$

These reactions generate hydroxyl radicals (2.73 V vs SHE, eq 1), ozone (2.31 V vs SHE, eqs 2 and 3), hydrogen peroxide (1.76 V vs SHE, eq 4), sulfate radicals (2.6 V vs SHE, eq 5), and peroxodisulfate ions (2.01 V vs SHE, eqs 6–8). These active species are primarily generated at the BDD electrode surface. In contrast, on the Pt electrode, hydroxyl radicals chemically interact with the surface to form platinum oxide species, which exhibit lower oxidation potential. This difference in the generation of active species and their oxidation potentials likely accounts for the observed variations in the product distribution between the BDD and Pt electrodes.

## Conclusions

This study demonstrated the successful electrochemical production of rare sugars through d-glucose oxidation using the BDD electrode. Comprehensive product analysis using dual labeling strategies (ABEE and l-tryptophan amide) confirmed the formation of various rare sugars including d-arabinose, d-erythrose, d-glyceraldehyde, and l-glyceraldehyde. Under optimized conditions using a BDD electrode, maximum concentrations of 0.126 mmol L^–1^ d-arabinose, 0.0544 mmol L^–1^ d-erythrose, and 0.148 mmol L^–1^ d, l-glyceraldehyde were achieved, significantly exceeding the yields obtained with conventional Pt electrodes.

The applied potential was identified as a critical parameter affecting product distribution and yield, with optimal rare sugar production observed at 2.5 V vs Ag/AgCl. This optimal potential represents a balance between sufficient oxidative power for glucose conversion and minimal product degradation. Mechanistic investigations suggest that rare sugar formation proceeds through multiple pathways, including sequential oxidation-decarboxylation reactions and carbon–carbon bond cleavage, facilitated by electrochemically generated active species at the BDD electrode surface.

The superior performance of BDD electrodes compared to Pt electrodes is attributed to their wide potential window, efficient generation of oxidizing species, and unique surface characteristics that prevent catalyst deactivation. These findings establish electrochemical oxidation using BDD electrodes as a promising approach for the selective synthesis of rare sugars from readily available glucose feedstock. This method offers advantages over conventional enzymatic and chemical approaches, particularly in terms of operational simplicity and potential scalability.

## Methods

### Electrochemical Decomposition of d-Glucose

Electrochemical experiments were conducted using a two-compartment three-electrode cell separated by an ion exchange membrane (Nafion NR212), as shown in Figure S6. Either a BDD (boron concentration: 5000 ppm, De Nora Permelec Ltd.) or Pt (Nilaco) electrode was used as the working electrode, while Pt served as the counter electrode. Both electrodes had an active area of 1.8 cm × 1.8 cm. The working electrode compartment contained 10 mL of 0.2 mol L^–1^ sodium sulfate solution (Wako Pure Chemical, 99.0%) with 10 mmol L^–1^ d-glucose (Wako Pure Chemical, 98.0%), while the counter electrode compartment contained 10 mL of 0.2 mol L^–1^ sodium sulfate solution. The electrochemical cell was maintained at 25 °C by using a cooling plate. Solution circulation was achieved using a quantitative liquid delivery pump (EYELA MP-4000) at a flow rate of 27.4 mL min^–1^. Prior to electrolysis, a 100 μL sample was collected for initial composition analysis. Electrolysis was then performed using a potentiostat (Meidensha HZ-7000) under either constant current (10 mA) or constant potential conditions. For constant potential experiments, applied potentials were set at 1.5, 2.0, 2.5, or 3.0 V vs Ag/AgCl, with electrolysis conducted for 6 h in each case.

### Qualitative and Quantitative Analysis of Products

Product analysis of the electrolyzed samples was performed using HPLC following *p*-aminobenzoic acid ethyl ester (ABEE) labeling.^46^ The ABEE labeling reagent was prepared by combining ABEE (Tokyo Chemical Industry, 99%), sodium cyanoborohydride (Tokyo Chemical Industry, 95%), and acetic acid (Wako Pure Chemical, 99.7%) in a molar ratio of 8:2:27. This mixture was dissolved in methanol (Wako Pure Chemical, 99.8%) to achieve a final volume nine times that of the combined reagents. For the labeling procedure, 10 μL of sample was mixed with 40 μL of ABEE labeling reagent, stirred for 30 s, and incubated at 80 °C for 1 h in a block bath (SCINICS DB105). After air cooling to room temperature for 5 min, 200 μL each of ultrapure water and chloroform (Wako Pure Chemical, 99.8%) were added and mixed for 1 min. The upper layer (150 μL) was collected, diluted with 300 μL of ultrapure water, and used for HPLC analysis.

Chromatographic analysis was performed using a HPLC system equipped with a UV detector (SPD-20A, Shimadzu) operating at 305 nm. Chromatographic separation was achieved using a reverse-phase column (CAPCELL PAK C18 AQ, 150 mm × 4.6 mm I.D., OSAKA SODA) maintained at 40 °C. The mobile phase consisted of 20 mmol L^–1^ ammonium acetate (Wako Pure Chemical, 97.0%) and acetonitrile (Wako Pure Chemical, 99.8%) at a ratio of 87:13 (v/v), delivered at a flow rate of 1.0 mL min^–1^.

Molecular weight determination of ABEE-labeled products was conducted using LC/MS (LC-MS8050, Shimadzu). The samples were diluted 10-fold prior to analysis. Separation was performed on a CAPCELL PAK C18 AQ column (150 mm × 2.0 mm I.D.) at 40 °C, with a flow rate of 0.2 mL min^–1^. The mobile phase composition was maintained at 87:13 (v/v) of 10 mmol L^–1^ ammonium acetate solution and acetonitrile. Mass spectrometric detection was performed using electrospray ionization in scan mode.

### l-Tryptophan Amide Labeling

The l-tryptophan amide labeling reagent was prepared by sequential addition of components:^36^ first, 2 mL of methanol was added to 4 mL of 100 mmol L^–1^ sodium tetraborate solution (Wako Pure Chemical, 99.5%), followed by addition of l-tryptophan amide to achieve a final concentration of 100 mmol L^–1^. The labeling reaction was performed by combining 150 μL of the prepared labeling reagent with 50 μL of sample and 50 μL of 2 mol L^–1^ dimethylamine borane solution (Wako Pure Chemical, 97%). The reaction mixture was incubated at 40 °C for 4 h in a block bath. Following incubation, 250 μL of 1 mol L^–1^ sodium chloride solution (Wako Pure Chemical, 99.5%) was added, and the mixture was diluted 4-fold with mobile phase for HPLC analysis.

Chromatographic analysis was performed using an HPLC system equipped with a UV detector (SPD-20A, Shimadzu) operating at 220 nm. Separation was achieved using a CAPCELL PAK C18 AQ reverse-phase column (150 mm × 4.6 mm I.D., OSAKA SODA) maintained at 40 °C. The mobile phase consisted of 10 mmol L^–1^ ammonium acetate buffer (pH 7.6) containing 1.5 mmol L^–1^ butylboronic acid (Tokyo Chemical Industry, 97%) and acetonitrile in a ratio of 95:5 (v/v), delivered at a flow rate of 1.0 mL min^–1^.

Molecular weight analysis of the l-tryptophan amide-labeled products was performed using LC/MS (LC-MS8050, Shimadzu) with electrospray ionization in scan mode. The chromatographic separation employed identical column type and mobile phase composition as the HPLC analysis, but with a modified flow rate of 0.6 mL min^–1^ at 40 °C to accommodate MS detection requirements.

### Qualitative and Quantitative Analysis of Organic Acids

The organic acids produced during the electrochemical oxidation of d-glucose were analyzed using HPLC (Shimadzu). Chromatographic separation was achieved using a Rezex ROA-Organic Acid H^+^ (8%) column (300 mm × 7.8 mm I.D., Phenomenex) maintained at 60 °C. The mobile phase consisted of 2.5 mmol L^–1^ sulfuric acid, delivered at a flow rate of 0.5 mL min^–1^. Detection was performed using a UV detector (SPD-20A, Shimadzu) operating at 210 nm.

## Supplementary Material



## Notes

Illustration of experimental setup and mass spectra of products (PDF)

## References

[1] Kobayashi T., Nakajima L. (2021). Sustainable development
goals for advanced materials
provided by industrial wastes and biomass sources. Curr. Opin. Green Sustainable Chem..

[2] Antar M., Lyu D., Nazari M., Shah A., Zhou X., Smith D. L. (2021). Biomass
for a sustainable bioeconomy: An overview of world biomass production
and utilization. Renewable Sustainable Energy
Rev..

[3] Mika L. T., Cséfalvay E., Németh Á. (2018). Catalytic conversion of carbohydrates
to initial platform chemicals: chemistry and sustainability. Chem. Rev..

[4] Yang M., Yuan Z., Peng R., Wang S., Zou Y. (2022). Recent progress
on electrocatalytic valorization of biomass-derived organics. Energy Environ. Mater..

[5] Zhang Q., Wan Z., Iris K., Tsang D. C. (2021). Sustainable production of high-value
gluconic acid and glucaric acid through oxidation of biomass-derived
glucose: A critical review. J. Cleaner Prod..

[6] Chen S. S., Yu I. K. M., Tsang D. C. W., Yip A. C. K., Khan E., Wang L., Ok Y. S., Poon C. S. (2017). Valorization of
cellulosic food waste into levulinic acid catalyzed by heterogeneous
Brønsted acids: Temperature and solvent effects. Chem. Eng. J..

[7] Chen X., Liu Y., Wu J. (2020). Sustainable production
of formic acid from biomass
and carbon dioxide. Mol. Catal..

[8] Demirbaş A. (2001). Biomass resource
facilities and biomass conversion processing for fuels and chemicals. Energy Convers. Manage..

[9] Granstrom T. B., Takata G., Tokuda M., Izumori K. (2004). Izumoring:
a novel
and complete strategy for bioproduction of rare sugars. J. Biosci. Bioeng..

[10] Hayashi N., Iida T., Yamada T., Okuma K., Takehara I., Yamamoto T., Yamada K., Tokuda M. (2010). Study on the
postprandial
blood glucose suppression effect of D-psicose in borderline diabetes
and the safety of long-term ingestion by normal human subjects. Biosci., Biotechnol., Biochem..

[11] Ochiai M., Nakanishi Y., Yamada T., Iida T., Matsuo T. (2013). Inhibition
by dietary D-psicose of body fat accumulation in adult rats fed a
high-sucrose diet. Biosci., Biotechnol., Biochem..

[12] Ishihara Y., Katayama K., Sakabe M., Kitamura M., Aizawa M., Takara M., Itoh K. (2011). Antioxidant
properties of rare sugar
D-allose: Effects on mitochondrial reactive oxygen species production
in Neuro2A cells. J. Biosci. Bioeng..

[13] Yamada K., Noguchi C., Kamitori K., Dong Y., Hirata Y., Hossain M. A., Tsukamoto I., Tokuda M., Yamaguchi F. (2012). Rare sugar
D-allose strongly induces thioredoxin-interacting protein and inhibits
osteoclast differentiation in Raw264 cells. Nutr. Res..

[14] Sakoguchi H., Yoshihara A., Shintani T., Okuma K., Izumori K., Sato M. (2016). Growth inhibitory effect of D-arabinose against the nematode Caenorhabditis
elegans: discovery of a novel bioactive monosaccharide. Bioorg. Med. Chem. Lett..

[15] Guo Y., Chen N., Zhao M., Cao B., Zhu F., Guo C., Shi Y., Wang Q., Li Y., Zhang L. (2024). D-arabinose
acts as antidepressant by activating the ACSS2-PPARγ/TFEB axis
and CRTC1 transcription. Pharmacol. Res..

[16] Beerens K., Desmet T., Soetaert W. (2012). Enzymes for
the biocatalytic production
of rare sugars. J. Ind. Microbiol. Biotechnol..

[17] Liu W., Wang P. (2007). Cofactor regeneration
for sustainable enzymatic biosynthesis. Biotechnol.
Adv..

[18] Wang Y., Carder H. M., Wendlandt A. E. (2020). Synthesis of rare sugar isomers through
site-selective epimerization. Nature.

[19] Mukaiyama T., Miwa T., Nakatsuka T. (1982). A Stereoselective
Synthesis of 2-Amino-2-Deoxy-d-Arabinose and d-Ribose. Chem.
Lett..

[20] Haworth W. N., Peat S., Whetstone J. (1938). 371. Descent of the series of methylated
sugars by the Weerman reaction. J. Chem. Soc..

[21] Gao D.-M., Kobayashi T., Adachi S. (2015). Production of rare sugars from common
sugars in subcritical aqueous ethanol. Food
Chem..

[22] van
der Ham M. P. J. M., van Keulen E., Koper M. T., Tashvigh A. A., Bitter J. H. (2023). Steering the selectivity of electrocatalytic glucose
oxidation by the Pt oxidation state. Angew.
Chem..

[23] Armstrong R. D., Hirayama J., Knight D. W., Hutchings G. J. (2019). Quantitative
determination of Pt-catalyzed D-glucose oxidation products using 2D
NMR. ACS Catal..

[24] Hay G. W., Smith F. (1969). Electrolysis of low
molecular weight carbohydrates in non-aqueous
media. i. the products of electrolysis of monosaccharides. Can. J. Chem..

[25] Xu J., Yokota Y., Wong R. A., Kim Y., Einaga Y. (2020). Unusual electrochemical
properties of low-doped boron-doped diamond electrodes containing
sp^2^ carbon. J. Am. Chem. Soc..

[26] Macpherson J. V. (2015). A practical
guide to using boron doped diamond in electrochemical research. Phys. Chem. Chem. Phys..

[27] Muzyka K., Sun J., Fereja T. H., Lan Y., Zhang W., Xu G. (2019). Boron-doped
diamond: current progress and challenges in view of electroanalytical
applications. Anal. Methods.

[28] Lee K.-M., Lee H.-J., Seo J., Lee T., Yoon J., Kim C., Lee C. (2022). Electrochemical oxidation
processes for the treatment
of organic pollutants in water: performance evaluation using different
figures of merit. ACS ES&T Eng..

[29] Moreira F. C., Boaventura R. A., Brillas E., Vilar V. J. (2017). Electrochemical
advanced oxidation processes: a review on their application to synthetic
and real wastewaters. Appl. Catal., B.

[30] Divyapriya G., Nidheesh P. V. (2021). Electrochemically
generated sulfate radicals by boron
doped diamond and its environmental applications. Curr. Opin. Solid State Mater. Sci..

[31] Fiorani A., Valenti G., Paolucci F., Einaga Y. (2023). Electrogenerated chemiluminescence
at boron-doped diamond electrodes. Chem. Commun..

[32] Yu H., Wang H., Quan X., Chen S., Zhang Y. (2007). Amperometric
determination of chemical oxygen demand using boron-doped diamond
(BDD) sensor. Electrochem. Commun..

[33] Nidheesh P. V., Divyapriya G., Oturan N., Trellu C., Oturan M. A. (2019). Environmental
Applications of Boron-Doped Diamond Electrodes: 1. Applications in
Water and Wastewater Treatment. ChemElectroChem.

[34] Espinoza L. C., Candia-Onfray C., Vidal J., Salazar R. (2021). Influence of the chemical
nature of Boron-Doped diamond anodes on wastewater treatments. Curr. Opin. Solid State Mater. Sci..

[35] Ganiyu S. O., dos Santos E. V., Martínez-Huitle C. A., Waldvogel S. R. (2022). Opportunities
and challenges of thin-film boron-doped diamond electrochemistry for
valuable resources recovery from waste: Organic, inorganic, and volatile
product electrosynthesis. Curr. Opin. Electrochem..

[36] Akabane M., Yamamoto A., Aizawa S.-I., Taga A., Kodama S. (2014). Simultaneous
enantioseparation of monosaccharides derivatized with L-tryptophan
by reversed phase HPLC. Anal. Sci..

[37] Chen X., Zhang Z., Yang Y., Hu B., Wu Q., Fan W., Hao J., Shi W. (2024). NiCu-based
catalysts with high selectivity
for electro–oxidation of glucose to formic acid. Chem. Eng. Sci..

[38] Liu W.-J., Xu Z., Zhao D., Pan X.-Q., Li H.-C., Hu X., Fan Z.-Y., Wang W.-K., Zhao G.-H., Jin S. (2020). Efficient electrochemical
production of glucaric acid and H_2_ via glucose electrolysis. Nat. Commun..

[39] Martínez-Huitle C. A., Rodrigo M. A., Sirés I., Scialdone O. (2015). Single and
coupled electrochemical processes and reactors for the abatement of
organic water pollutants: a critical review. Chem. Rev..

[40] Bao H., Wu M., Meng X., Han H., Zhang C., Sun W. (2023). Application
of electrochemical oxidation technology in treating high-salinity
organic ammonia-nitrogen wastewater. J. Environ.
Chem. Eng..

[41] Kondo T., Tamura Y., Hoshino M., Watanabe T., Aikawa T., Yuasa M., Einaga Y. (2014). Direct determination of chemical
oxygen demand by anodic decomposition of organic compounds at a diamond
electrode. Anal. Chem..

[42] Kuczyński M., Łuba M., Mikołajczyk T., Pierożyński B. (2023). The influence
of resorcinol on the kinetics of underpotentially deposited hydrogen,
cathodic hydrogen and anodic oxygen evolution reactions, examined
at polycrystalline Pt electrode in 0.1 M NaOH solution. Int. J. Hydrogen Energy.

[43] Einaga Y. (2010). Diamond electrodes
for electrochemical analysis. J. Appl. Electrochem..

[44] Chou C., Chou T.-C. (2003). Paired electrooxidation
IV. Decarboxylation of sodium
gluconate to D-arabinose. J. Appl. Electrochem..

[45] Moggia G., Kenis T., Daems N., Breugelmans T. (2020). Electrochemical
Oxidation of D-Glucose in Alkaline Medium: Impact of Oxidation Potential
and Chemical Side Reactions on the Selectivity to D-Gluconic and D-Glucaric
Acid. ChemElectroChem.

[46] Yasuno S., Murata T., Kokubo K., Yamaguchi T., Kamei M. (1997). Two-mode analysis by high-performance
liquid chromatography of ρ-aminobenzoic
ethyl ester-derivatized monosaccharides. Biosci.,
Biotechnol., Biochem..

