# Supplementary material for: Generation of Rare Sugars by Electrochemical Oxidation of d‑Glucose Using Boron-Doped Diamond Electrode
Source: J Am Chem Soc. 2025 May 8;147(25):21363–74. doi: 10.1021/jacs.4c17553 (PMC12203589; doi:10.1021/jacs.4c17553)
Supplement: Supplementary file 1 [file ja4c17553_si_001.pdf]

Supporting information

Generation of Rare Sugars by Electrochemical Oxidation of D-Glucose Using Boron-Doped Diamond Electrode

Kio Kawakatsu[a], Sho Usuki[a]*, Tiangao Jiang[a], Naoko Taki[a], Yuma Uesaka[a], Haru Togawa[a], Shanhu Liu[b], Yasuaki Einaga[c]*, Kazuya Nakata[a]*

[a]Graduate School of Bio-Applications and Systems Engineering, Tokyo University of Agriculture and Technology, 2-24-16 Naka-cho, Koganei, Tokyo 184-0012, Japan
[b]Henan Joint International Research Laboratory of Environmental Pollution Control Materials, Henan Key Laboratory of Polyoxometalate Chemistry, College of Chemistry and Chemical Engineering, Henan University, Kaifeng, 475004, PR China
[c]Department of Chemistry, Keio University, 3-14-1 Hiyoshi, Yokohama 223-8522, Japan
* Email: fv7248@go.tuat.ac.jp; Phone: +81-42-388-7790
* Email: einaga@chem.keio.ac.jp; Phone: +81-45-566-1704
* Email: nakata@go.tuat.ac.jp; Phone: +81-42-388-7767

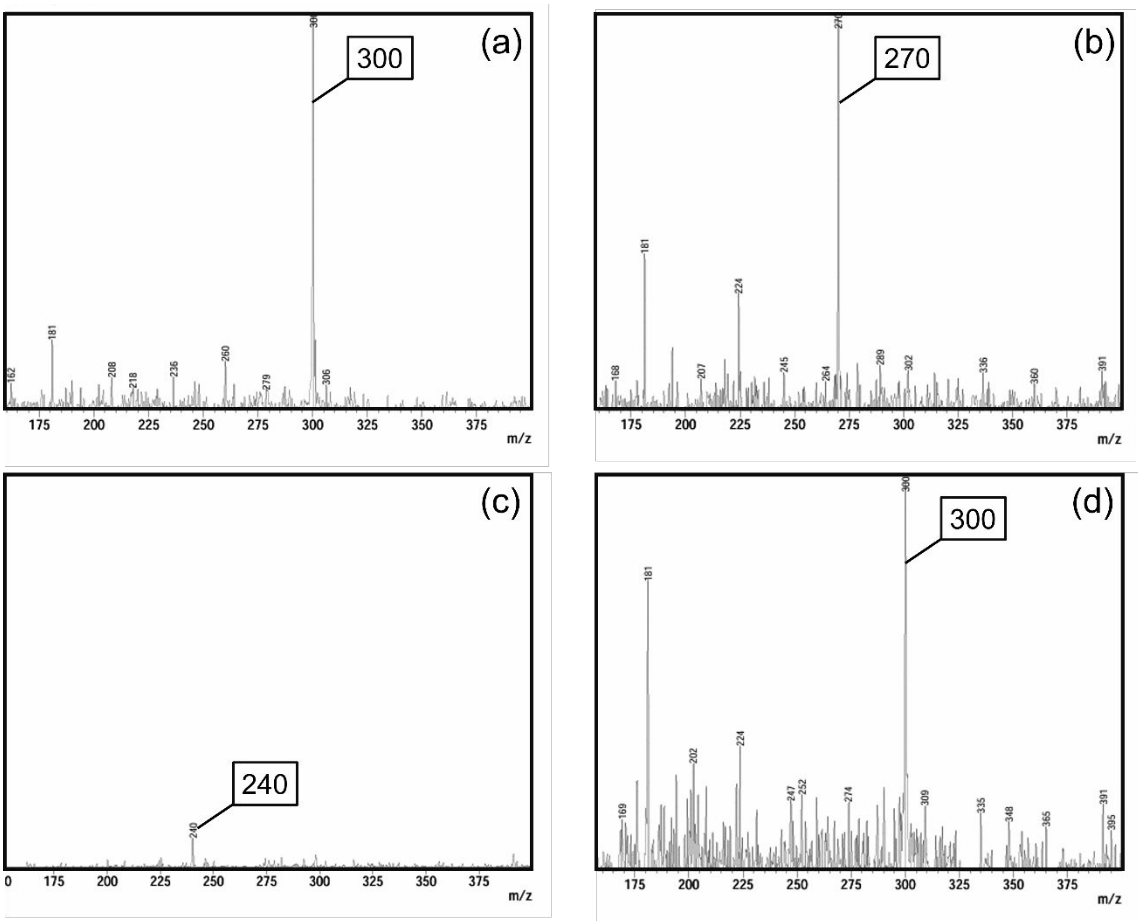

Figure S1. Mass spectra of the ABEE-labeled product found at R.T. = (a) 21.3 min, (b) 25.6 min, and (c) 38.3 min in HPLC analysis, obtained from 2 h electrolysis using BDD electrode. (d) Mass spectrum of the ABEE-labeled product found at R.T. = 21.2 min in the HPLC analysis, obtained after 6 h of electrolysis using a Pt electrode. Experimental conditions: volume = 20 mL (anode: 10 mL, cathode: 10 mL), electrode area = 18 mm × 18 mm, cathode = Pt, electrolyte = 0.2 mol L^{-1} Na_2SO_4, initial concentration of glucose = 10 mmol L^{-1}.

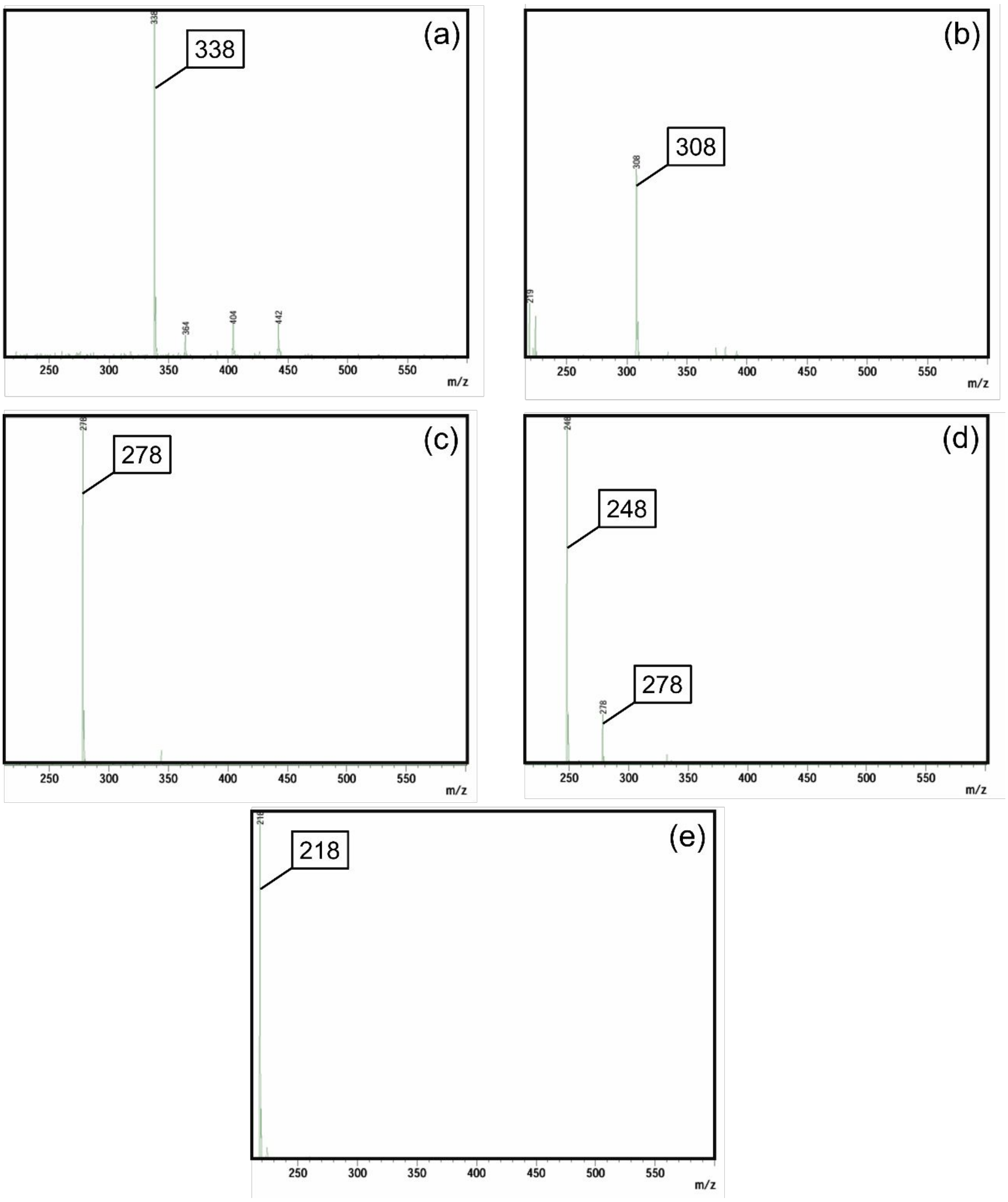

Figure S2. Mass spectra of the L-tryptophanamide-labeled product found at R.T. = (a) 79.4 min, (b) 37.3 min, (c) 40.6 min, (d) 45.0 min, and (e) 31.2 min in HPLC analysis, obtained from 2 h electrolysis using BDD electrode. Experimental conditions: volume = 20 mL (anode: 10 mL, cathode: 10 mL), electrode area = 18 mm × 18 mm, cathode = Pt, electrolyte = 0.2 mol L^{-1} Na_2SO_4, initial concentration of glucose = 10 mmol L^{-1}.

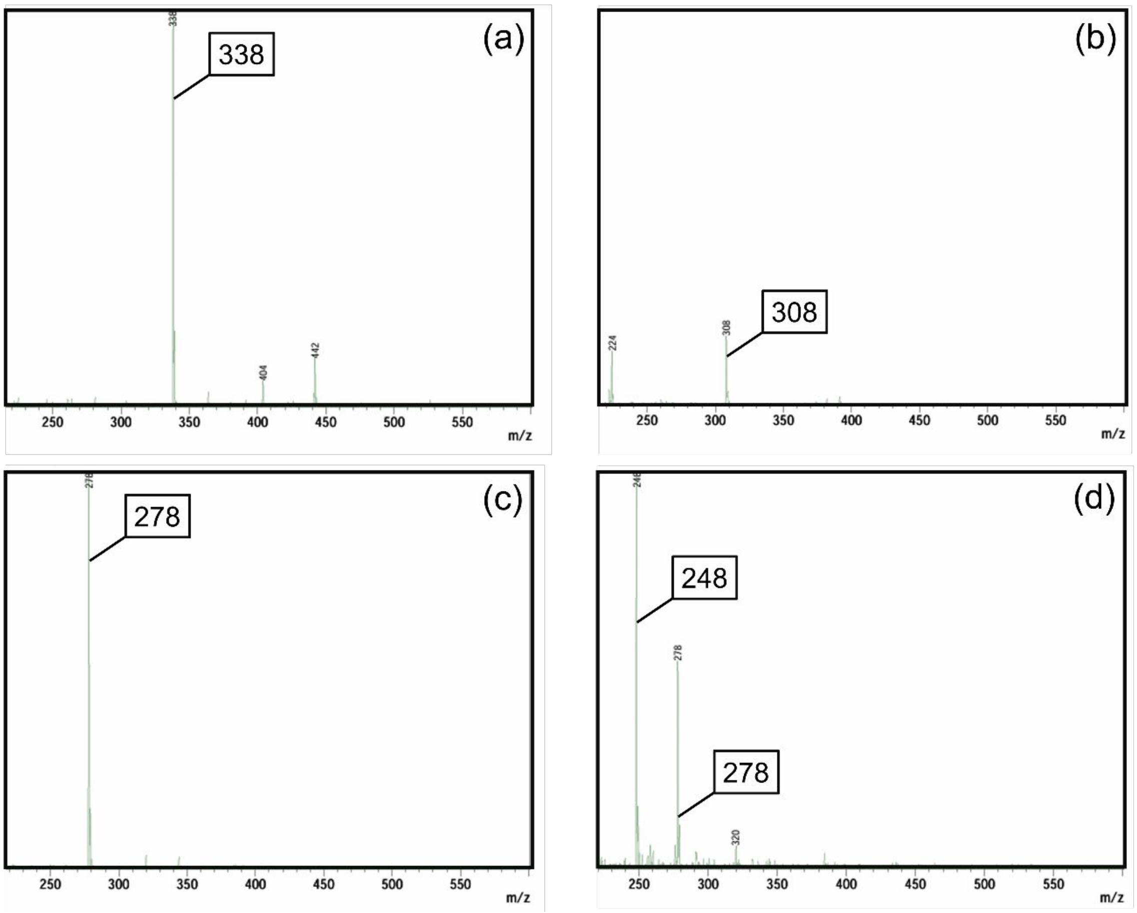

Figure S3. Mass spectrum of the L-tryptophanamide-labeled product found at R.T. = (a) 79.3 min, (b) 37.3 min, (c) 40.6 min, and (d) 45.1 min in HPLC analysis, obtained from 6 h electrolysis using Pt electrode. Experimental conditions: volume = 20 mL (anode: 10 mL, cathode: 10 mL), electrode area = 18 mm × 18 mm, cathode = Pt, electrolyte = 0.2 mol L^{-1} Na_2SO_4, initial concentration of glucose = 10 mmol L^{-1}.

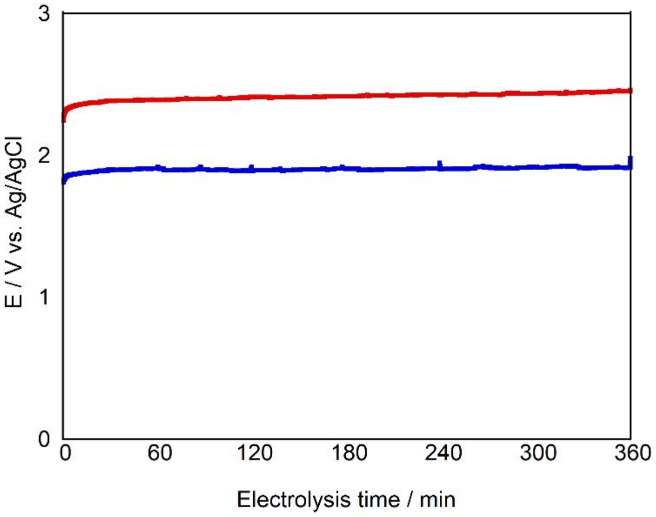

Figure S4. Time-dependent changes in electrode potential during constant current electrolysis of D-glucose using BDD (blue) and Pt (red) anodes. Experimental conditions: volume = 20 mL (anode: 10 mL, cathode: 10 mL), electrode area = 18 mm × 18 mm, cathode = Pt, electrolyte = 0.2 mol L^{-1} Na_2SO_4, initial concentration of glucose = 10 mmol L^{-1}, applied current density = 10 mA cm^{-2}.

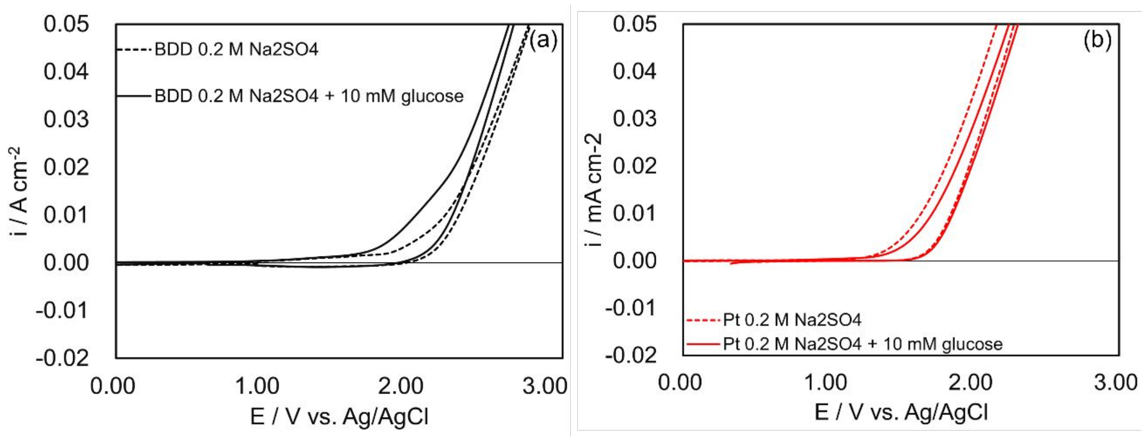

Figure S5. CV of (a) BDD and (b) Pt electrode with (solid line) and without (dotted line) 10 mmol L^{-1} glucose. Experimental conditions: volume = 20 mL (anode: 10 mL, cathode: 10 mL), electrode area = 18 mm × 18 mm, cathode = Pt, electrolyte = 0.2 mol L^{-1} Na_2SO_4.

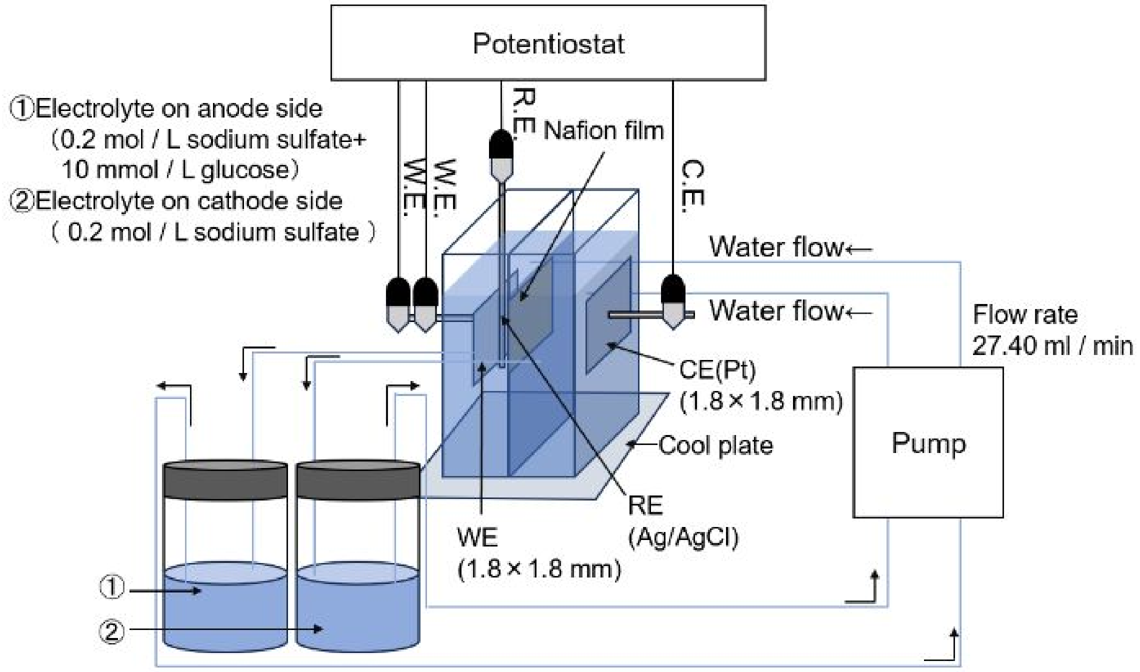

Figure S6. Schematic illustration of the two-compartment electrochemical cell setup for D-glucose oxidation.

Table S1. Faradaic efficiency of BDD and Pt after 6 hours electrolysis[†]

Anode	BDD	Pt
D-arabinose	0.6	0.05
D-erythrose	0.6	n.d.
D,L-glyceraldehyde	0.2	n.d.
Gluconic acid	0.5	0.7
Formic acid	2.2	0.1
Carbon dioxide	43	n.d.
Total	47	0.8

n.d.: below detection limit (< 0.01%)

[†]The Faradaic efficiencies presented here were calculated based on specific assumptions about electron transfer requirements: 1-electron for arabinose, 2-electron for erythrose, 3-electron for glyceraldehyde, and 1-electron for formic acid, gluconic acid, and CO_2. In actual reactions, particularly for the formation of formic acid and CO_2, various reaction pathways involving 1 to 6 electrons may occur. Given these complex reaction pathways, please note that the values in this table are based on calculations that simplify the complexity of the actual reactions.

Table S2. Carbon balance distribution (%) during D-glucose electrolysis with BDD electrode

	1 h	2 h	3 h	4 h	5 h	6 h
D-glucose	71.8	47.8	30.5	17.8	9.0	4.1
D-arabinose	0.7	1.1	1.0	0.7	0.4	0.2
D-erythrose	0.2	0.4	0.4	0.3	0.2	0.1
D,L-glyceraldehyde	0.4	0.7	0.8	0.7	0.4	0.3
Gluconic acid	4.1	6.5	7.9	8.3	7.4	2.9
Formic acid	4.3	8.5	11.6	13.1	12.6	9.8
Carbon dioxide	36.2	69.5	99.1	126.9	144.5	164.1
Total	117.7	134.5	151.2	167.7	174.4	181.4

Table S3. Carbon balance distribution (%) during D-glucose electrolysis with Pt electrode

	1 h	2 h	3 h	4 h	5 h	6 h
D-glucose	99.5	97.2	96.5	94.6	93.2	90.1
D-arabinose	< 0.1	0.1	0.2	0.2	0.3	0.3
D-erythrose	n.d.	< 0.1	< 0.1	< 0.1	< 0.1	< 0.1
D,L-glyceraldehyde	n.d.	< 0.1	< 0.1	< 0.1	< 0.1	< 0.1
Gluconic acid	1.2	2.0	2.7	3.4	4.0	5.8
Formic acid	n.d.	0.1	0.1	0.1	0.2	0.2
Carbon dioxide	n.d.	n.d.	n.d.	n.d.	n.d.	n.d.
Total	100.7	99.5	99.6	98.4	97.6	96.5

n.d.: below detection limit (< 0.05%)
